# Utility of a novel activity monitor assessing physical activities and sleep quality in cats

**DOI:** 10.1371/journal.pone.0236795

**Published:** 2020-07-31

**Authors:** Atsushi Yamazaki, Kazuya Edamura, Koji Tanegashima, Yuma Tomo, Makoto Yamamoto, Hidehiro Hirao, Mamiko Seki, Kazushi Asano

**Affiliations:** 1 Laboratory of Veterinary Surgery, Department of Veterinary Medicine, College of Bioresource and Sciences, Nihon University, Fujisawa, Kanagawa, Japan; 2 Japan Animal Referral Medical Center, Inc., Kawasaki, Kanagawa, Japan; University of Lincoln, UNITED KINGDOM

## Abstract

Osteoarthritis in cats is more prevalent because cats are living longer with advancement in veterinary medicine. Objective evaluation of behavioral changes in cats with osteoarthritis can facilitate an early diagnosis. The objective of this study was to investigate the utility of a novel activity monitor for analyzing physical activities and sleep quality in cats. First, a novel activity monitor (Plus Cycle^®^; JARMeC, Kanagawa, Japan), with a built-in three-directional accelerometer and an air pressure sensor specifically designed for cats, was compared to a human activity monitor (Actical^®^; Philips Respironics, OR, USA) previously used in cats (n = 10). Second, the validity of the measurement accuracy of the amount of physical activity, the number of vibrations, the number of jumps, and the resting and sleeping time was evaluated using Plus Cycle^®^ in healthy cats (n = 6). Finally, the effects of gender and age of cats and time of day on the amount of physical activity, the number of vibrations, the number of jumps, and the resting and sleeping time were investigated in client-owned cats (n = 61). There were strong correlations between Plus Cycle^®^ and Actical^®^ in total activity (p < 0.05) and activity intensity (p < 0.05). When the physical activities were measured using Plus Cycle^®^ in healthy cats, those data were quantified with high accuracy. In addition, it was also found to be very accurate in discriminating the resting and sleeping conditions of cats. In client-owned cats, there were no significant differences with respect to gender in any measured traits. The amount of physical activity and the number of jumps significantly decreased with the age of the cat. In contrast, the resting and sleeping times significantly increased with the age of the cat. In conclusion, Plus Cycle^®^ can accurately and objectively assess physical activities and sleep quality with age of the cat, suggesting that this novel activity monitor can be used to manage the feline musculoskeletal health.

## Introduction

Cats are one of main patients visiting animal hospital and are living for longer with development of veterinary medicine. From this background, the cats with musculoskeletal disorders such as osteoarthritis (OA) are increasing in number. It was reported that 91 of 100 randomly selected cats aged 6 months to 20 years old, had at least one appendicular joint with radiographic OA [1]. Another study reported that 61% of cats aged 6 years and older and 82% of cats aged 14 years and older, had OA in at least one joint. In addition, a positive correlation between age and the presence of OA was shown by regression analysis [2].

As mentioned above, epidemiological studies that screened radiographs showed many elderly cats have OA, but the consultation rate of cats with OA tends to be low in the present situation. Unlike dogs, the most common clinical sign of OA in cats is not lameness [3]. Therefore, the owners of the cat cannot recognize the clinical signs associated with OA easily and are not aware that their own cat has OA. It has been reported that cats with OA have various other behavioral changes, but not lameness. In cats, the most common changes noted in association with OA are a reduced ability to jump (in 71%) and a reduced height of jump (in 67%) [3]. Other behavioral changes related to feline OA included: reduction in overall activity levels, resting more than usual, slow or stiff movements, increased sleeping time, difficulty in going up or down the stairs, difficulty in getting in and out of the litter box, reduction or difficulty with grooming, claw overgrowth, resentment of handling, reduced interaction with other pets and people, and loss of appetite [3–5].

To achieve early detection of OA based on clinical signs in cats, a subjective scoring system has been developed. To date, feline musculoskeletal pain index (FMPI), client-specific outcome measure (CSOM), Montreal instrument for cat arthritis testing for use by caretaker (MI-CAT(C)), Montreal instrument for cat arthritis testing for use by veterinarian (MI-CAT(V)) have been reported [6–9]. It may be difficult to obtain consistent opinions using those evaluations because the results of subjective evaluations are vague. To overcome this, an objective measurement method for evaluating the behavioral changes in cats with OA was needed.

Recently, the activity monitors with built-in accelerometer have attracted particular attention for early diagnosis of feline OA. These devices can record the changes in acceleration that relate to the intensity, frequency, duration of movement, and pattern of activity [10]. There has been only one report on assessing the measurement accuracy of activity monitor in cats, and the correlation between data generated by an accelerometer-based activity monitor and throughout distance moved was investigated [11]. The study documented that there was a strong correlation between activity data and moving distance of cats [11]. However, the activity monitor used in that study was the device developed for humans, not for animals, and had only a two-directional accelerometer. Thus, the device cannot measure the reduction in the number of jumps and sleeping time, which are the frequent signs of OA in cats [3]. From this background, we developed an activity monitor designed for dogs and cats, which has a built-in three-directional accelerometer and an air pressure sensor and comes with software to analyze sleeping time.

The objective of this study was to compare our new custom-developed device for cats and dogs with the human activity monitor previously reported in cats and to evaluate the validity of the measurement accuracy of physical activities and resting and sleeping times in cats. Furthermore, the effects of gender, age of cats and time of day (daytime or night-time) on physical activities and resting and sleeping times were investigated to collect basic data for the early diagnosis of feline OA.

## Materials and methods

### Comparison of Plus Cycle^®^ with human activity monitor previously reported in cats

Ten healthy cats, over one-year-old, belonging to the staff of Japan Animal Referral Medical Center (JARMeC), were recruited to participate in the present study. The study protocol was approved by the institutional animal care and use ethics committee (Protocol ID: F0602-17001) and written owner consent was obtained.

The activities of cats were monitored by two non-invasive activity monitors; Actical^®^ (Philips Respironics Inc., Bend, OR, U.S.A.) and Plus Cycle^®^ (JARMeC, Kanagawa, Japan). Actical^®^ was widely used to evaluate not only human activities but also canine activities [12–14]. In addition, Actical^®^ was also used to measure feline physical activity in a previous study [11]. The size and weight of Actical^®^ is 28×27×10 mm and 17 g, respectively.

Plus Cycle^®^ is an activity monitor newly developed specifically for measuring the activity of dogs and cats and has a built-in three-directional accelerometer and an air pressure sensor. The size and weight of Plus Cycle^®^ is diameter of 27 mm, thickness of 9.1 mm and 9 g, respectively.

In the present study, both the devices were attached to a single nylon collar on the subject cats. The accelerometer epochs (period of time where the device measure activity counts prior to saving it) were set at 1 minute. Data was collected while cats were engaging in their typical activities.

To access the data of Actical^®^, the device was removed from the collar, and then connected to a computer using the proprietary Actical^®^ reader device (ActiReader, Philips Respironics Inc., Bend, OR, U.S.A.). Data was downloaded using the Actical^®^ software. To access the data of Plus Cycle^®^, the device and the owner’s smartphone were paired through Bluetooth connectivity. Data was uploaded to the Plus Cycle^®^ application data server. When the data were analyzed, data was downloaded from the server to a computer. Activity data was measured by accelerometer and evaluated over an entire 24 hours period. Total activity data evaluated by Actical^®^ or Plus Cycle^®^ were defined as the total activity count during this time period. Activity intensity evaluated by Actical^®^ or Plus Cycle^®^ were defined as the activity counts for each minute.

### Validity of measurement accuracy of Plus Cycle^®^ in cats

Six healthy cats were recruited to participate as volunteers for the present study. This study was approved by the clinical research and trial ethics committee, Animal Medical Center, Nihon University (ANMEC-2019-03). All owners who participated in the present study consented to the collection of data.

**Fig 1:**
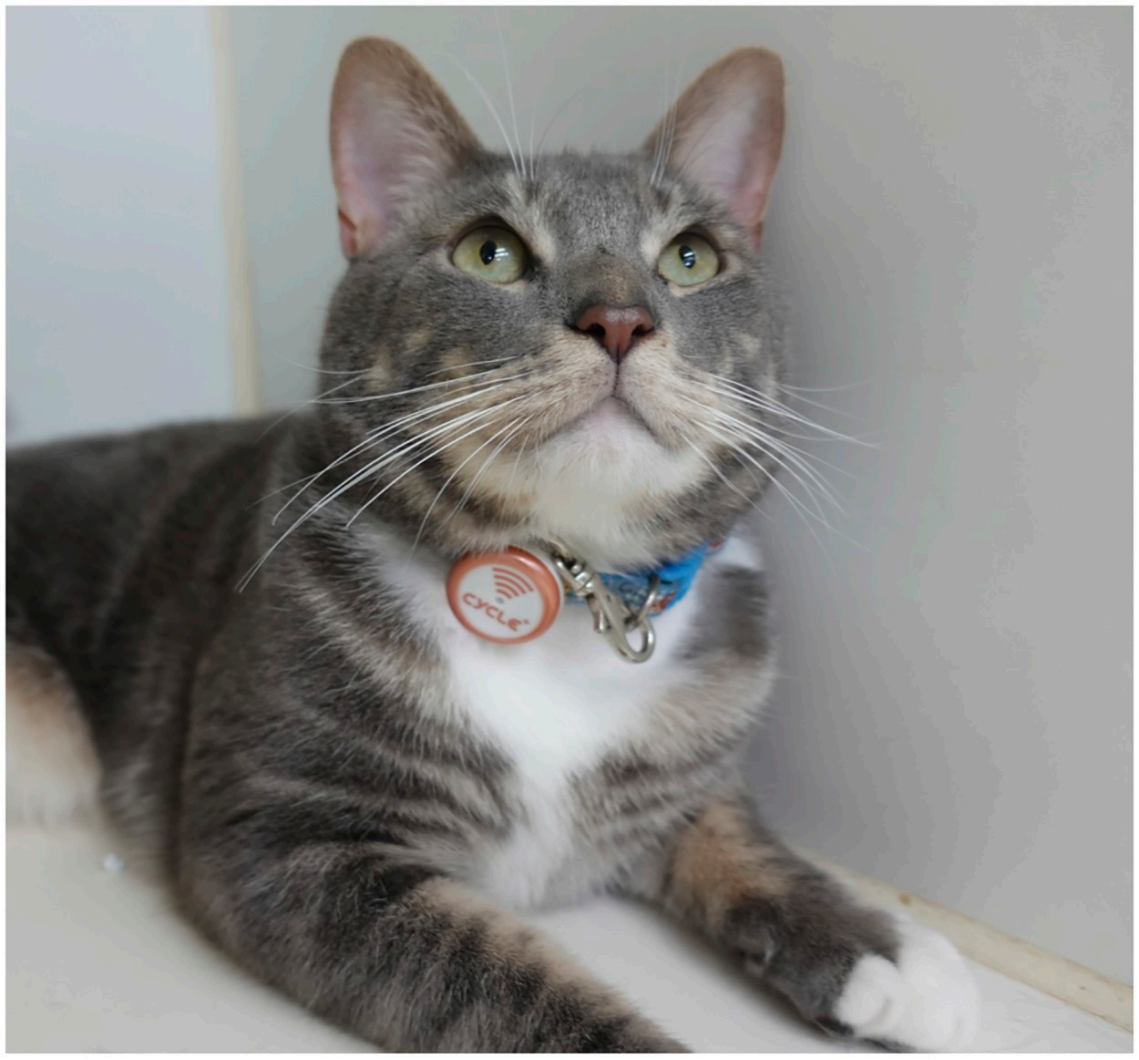
Method for mounting Plus Cycle^®^ to cats. The device was attached to a neck collar of the subject cats.

Plus Cycle^®^ was attached to a neck collar of the subject cats (Fig 1) and the amount of physical activity, the number of vibrations, the number of jumps, and resting and sleeping time were measured. Epochs of the accelerometer were set at 1 minute. The number of vibrations was defined as the number of detected accelerations per minute. In addition, the number of jumps was measured by an air pressure sensor and was counted when an air pressure change of 40 cm or more was detected. This activity data and actual movement of cats were compared, and the measurement accuracy of the Plus Cycle^®^ was evaluated.

**Fig 2:**
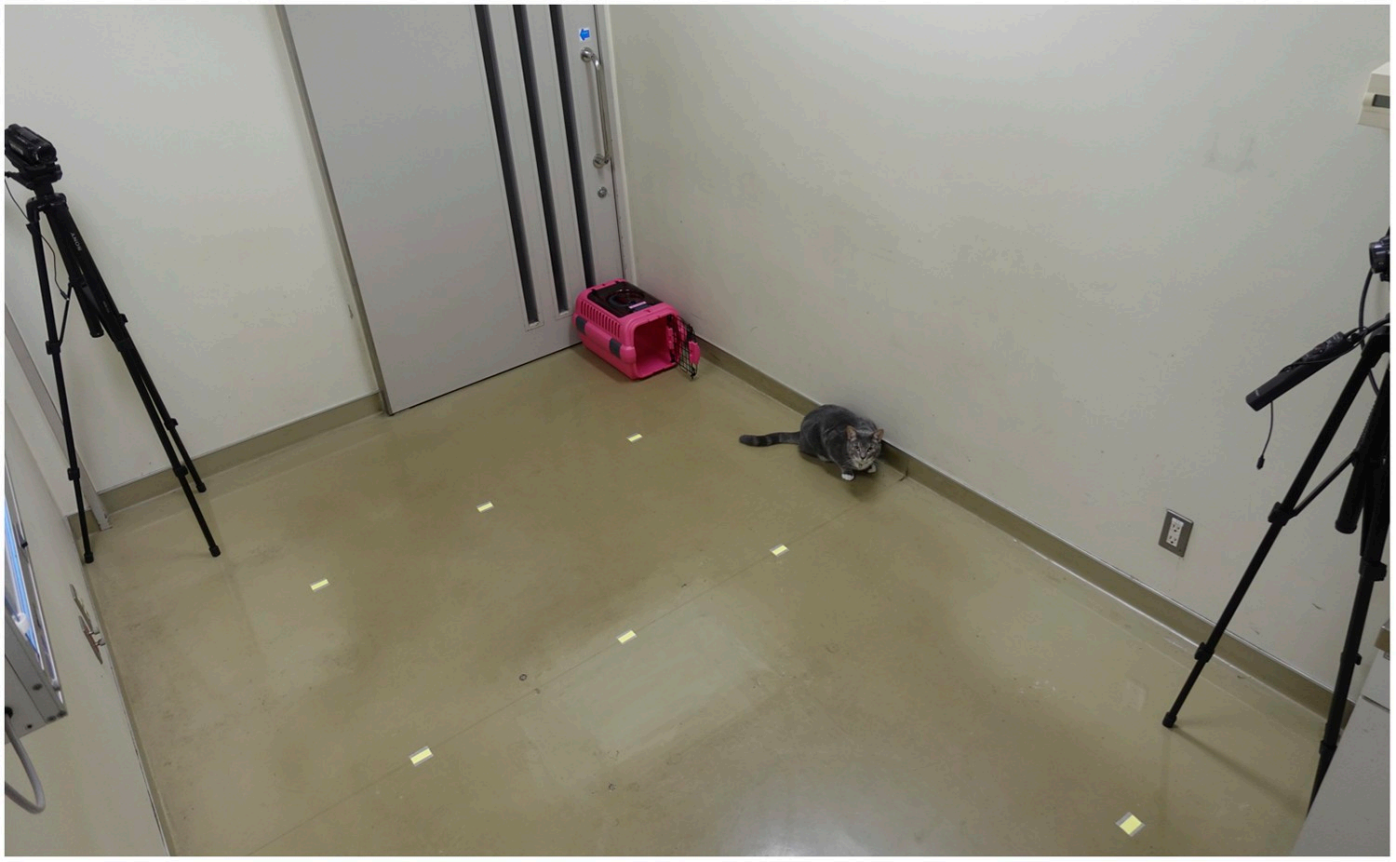
Verification method of the measurement accuracy of the amount of physical activity and the number of vibrations. Two video cameras were set diagonally in the room and recorded the behavior of cats. Then, the correlation between the activity data measured by Plus Cycle^®^ and recorded behavior of cats was examined.

To validate the accuracy of measuring the amount of physical activity and the number of vibrations, cats spent time freely in a room (3.6 m × 2.4 m) for an hour. Two video cameras were set diagonally in the room and recorded the behavior of cats (Fig 2). The moving time and distance were measured each minute by using video camera data. Downloaded activity data from Plus Cycle^®^ was imported into an excel spreadsheet from the raw file and compared with the video analysis data. Correlation between the activity data and moving time and distance was examined.

**Fig 3:**
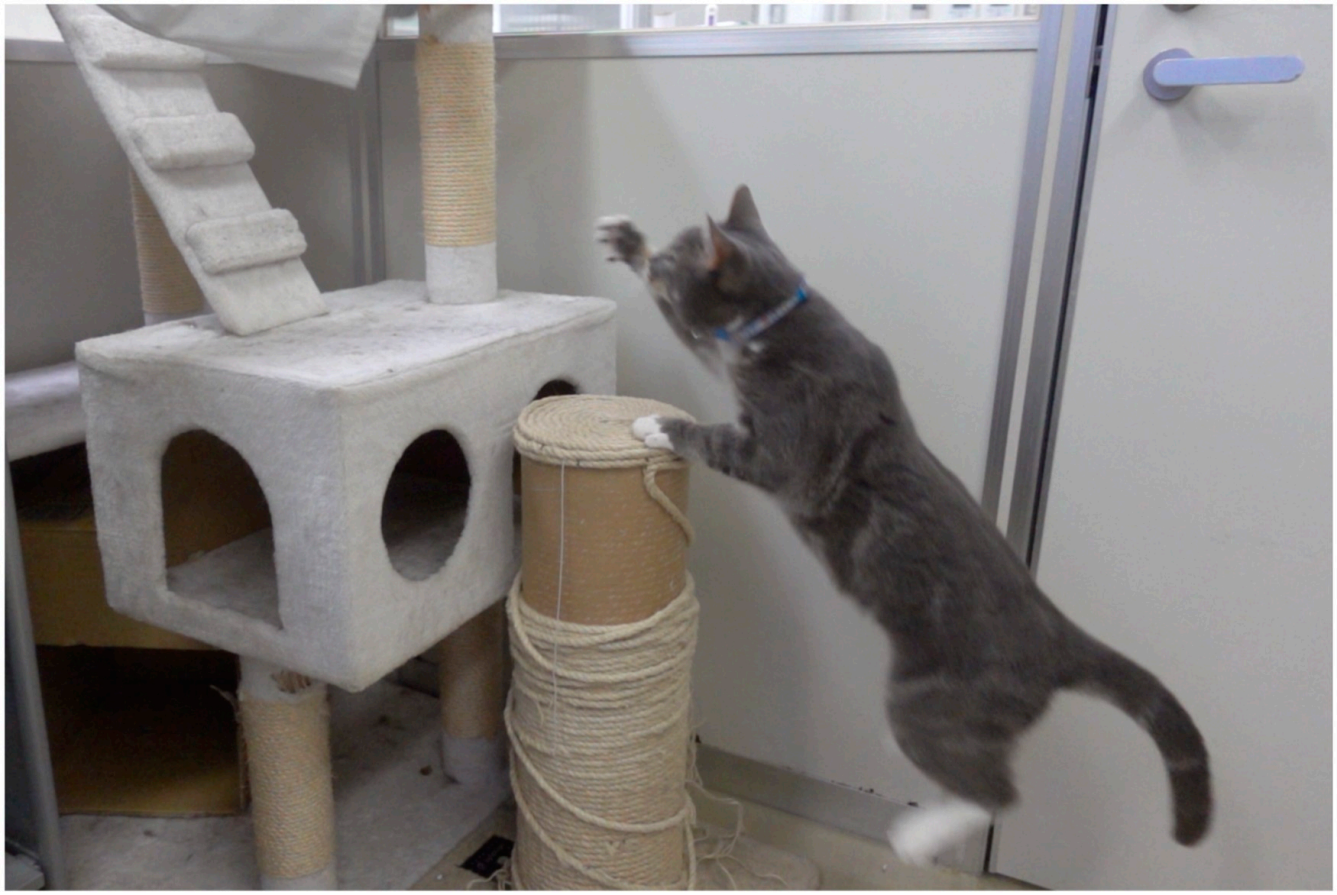
Verification method of the measurement accuracy of the number of jumps. Cats jumped up a step of over 40 cm and then the number of jumps counted by the observer and the number of jumps counted by Plus Cycle^®^ were compared.

To validate the accuracy of measuring the number of jumps, cats jumped up a step of over 40 cm (Fig 3). Then, the number of jumps counted by the observer and the number of jumps counted by Plus Cycle^®^ were compared.

**Fig 4:**
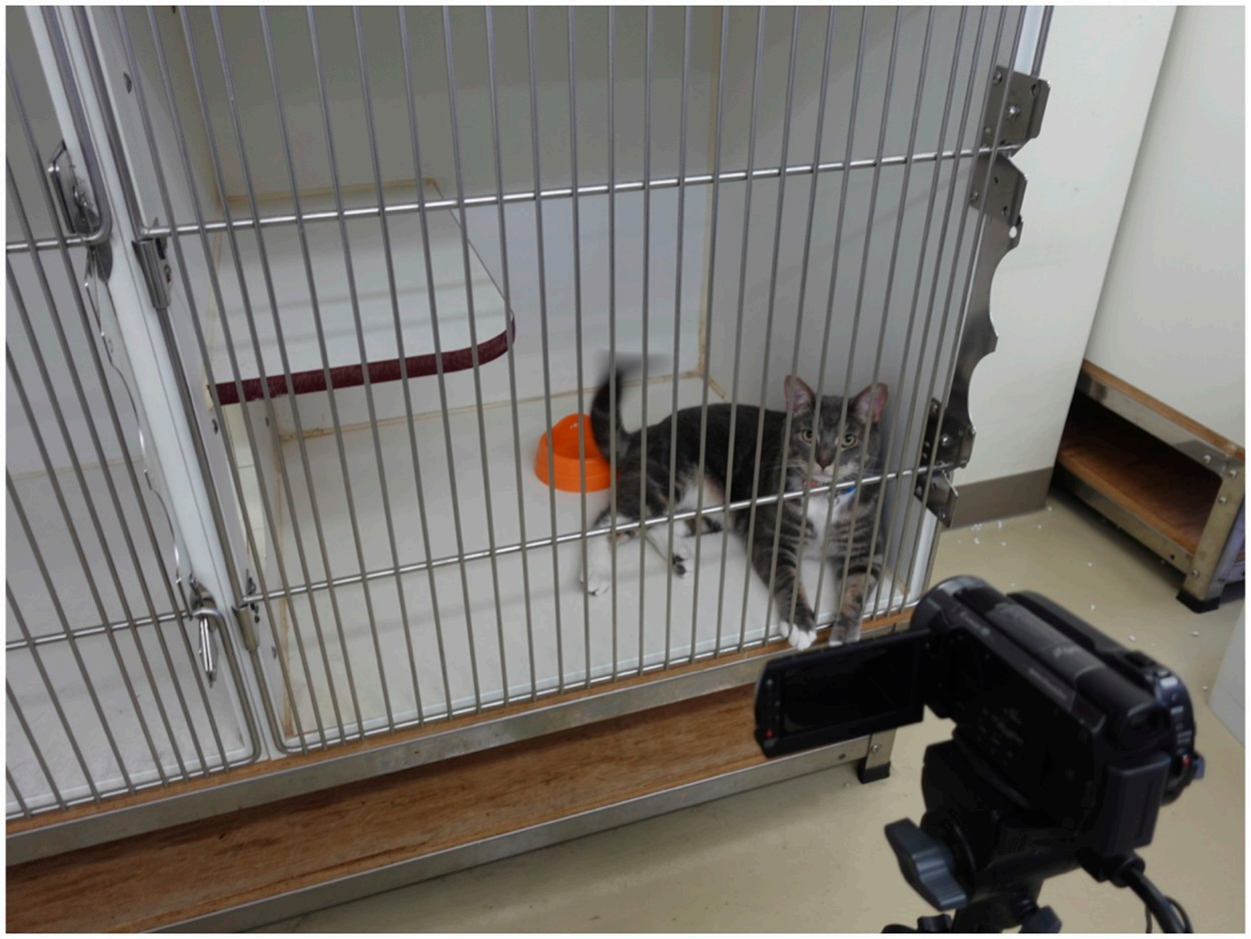
Verification method of the measurement accuracy of the resting and sleeping time. The behavior of cats was recorded by video camera from 00:00 to 08:00. Then, actual behavior of cats and the activity data measured by Plus Cycle^®^ were compared.

To validate the accuracy of measuring the resting and sleeping time, the behavior of cats was recorded by video camera from 00:00 to 08:00 (Fig 4). The feline behavior such as resting, sleeping, or awaking was recorded each minute. Awaking included activities such as standing, walking, playing and drinking water. Then, actual behavior of cats was recorded by an observer and activity data measured by Plus Cycle^®^ were compared.

### Assessment of physical activities and sleep quality using Plus Cycle^®^ in client-owned cats

The client-owned cats over 1 year of age were recruited to participate as volunteers in the present study. This study was approved by the clinical research and trial ethics committee, Animal Medical Center, Nihon University (ANMEC-2019-03). All owners who participated in the present study consented to the collection of data.

Plus Cycle^®^ was attached to a neck collar of the cats for 3 weeks. The amount of physical activity, the number of vibrations, the number of jumps, and resting and sleeping time were measured. The daily average values were calculated based on the obtained data. The effects of gender, age of cats and time of day (daytime or night-time) on physical activities and resting and sleeping times were investigated. The resting and sleeping time was defined as cumulative value of time when both the amount of physical activity and the number of vibrations of cats were zero per minute. In the present study, 06:00 to 23:59 was defined as daytime and 00:00 to 05:59 as night-time.

### Statistical analysis

All data were presented as mean ± standard deviation. Statistical analyses were performed using a data analysis software package (GraphPad Prism version 6.0 for Macintosh, GraphPad Software Inc., San Diego, California, USA). Pearson’s correlation coefficients were calculated to assess for correlation between the outputs of Plus Cycle^®^ and those of Actical^®^, and between moving time and distance. The relationship between age and outputs of each measured traits by Plus Cycle^®^ was also evaluated using Pearson’s correlation coefficients. The Mann-Whitney test was used for comparisons between the number of jumps counted by Plus Cycle^®^ and that counted by an observer. The differences of outputs of each measured trait by gender were also assessed using the Mann-Whitney test. To distinguish output data of rest and sleep or awake, the optimal cut off value of the amount of activity and the number of vibrations was decided using receiver operating characteristic (ROC) curve. Then, sensitivity, specificity and area under the receiver operating characteristic curve (AUC) were calculated to evaluate the accuracy that distinguish condition of rest and sleep or awake. Differences were considered significant when the *p*-value was less than 0.05.

## Results

### Comparison of Plus Cycle^®^ with human activity monitor previously reported in cats

The mean age and body weight of the 10 cats in the present study were 5.0 ± 2.2 years (range, 3 to 10 years) and 4.6 ± 1.4 kg (range, 2.7 to 6.8 kg), respectively. There were 3 neutered males and 7 spayed females. Breeds included Japanese domestic cats (n = 7) and European Shorthair (n = 3) (S1 Table).

**Fig 5:**
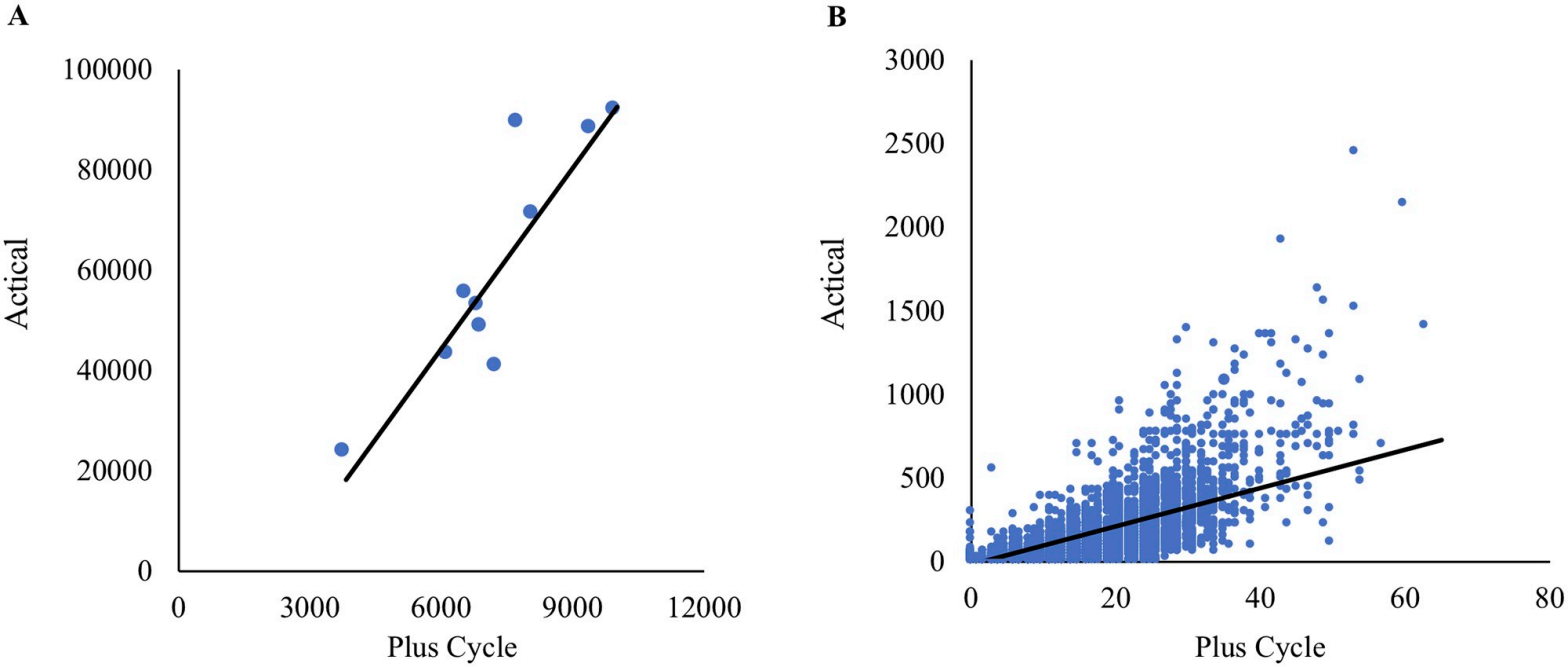
Correlation between output data of Actical^®^ and Plus Cycle^®^. Plots showing the correlation between Actical^®^ and Plus Cycle^®^ in total activity (A), or activity intensity (B).

The data during 1,440 minutes were collected simultaneously with both Actical^®^ and Plus Cycle^®^. This resulted in 28,800 total time points for comparison. Output data is summarized in Table 1. When evaluating the total activity data, there was a very strong correlation between the Actical^®^ and Plus Cycle^®^ (p < 0.05, r = 0.89). All data on activity intensity also had a strong correlation between the Actical^®^ and Plus Cycle^®^ (p < 0.05, r = 0.78) at all-time points (Fig 5). The correlation for individual cats ranged from 0.75 to 0.84.

**Table 1:** Summary of output data for total activity data and activity intensity.

	Total activity		Activity intensity	
	Plus Cycle^®^	Actical^®^	Plus Cycle^®^	Actical^®^
Minimum	3820.00	23651.00	0.00	0.00
Maximum	10012.00	91664.00	63.00	2455.00
Mean	7320.10	60238.00	5.08	41.83
S.D.	1734.38	23422.14	8.79	128.97
Correlation	0.89		0.78	

Total activity, the total activity count for 24 hours period evaluated by Actical^®^ or Plus Cycle^®^; Activity intensity, the activity counts for each minute evaluated by Actical^®^ or Plus Cycle^®^; Correlation, correlation coefficients showing correlation between outputs of Actical^®^ and Plus Cycle^®^; S.D., standard deviation.

### Validity of measurement accuracy of Plus Cycle^®^ in cats

Mean age and body weight of six cats in the present study were 3.5 ± 2.1 years (range, 1 to 6 years) and 4.7 ± 0.5 kg (range, 4.1 to 5.4 kg), respectively. All cats were neutered male Japanese domestic cats (S2 Table).

**Fig 6:**
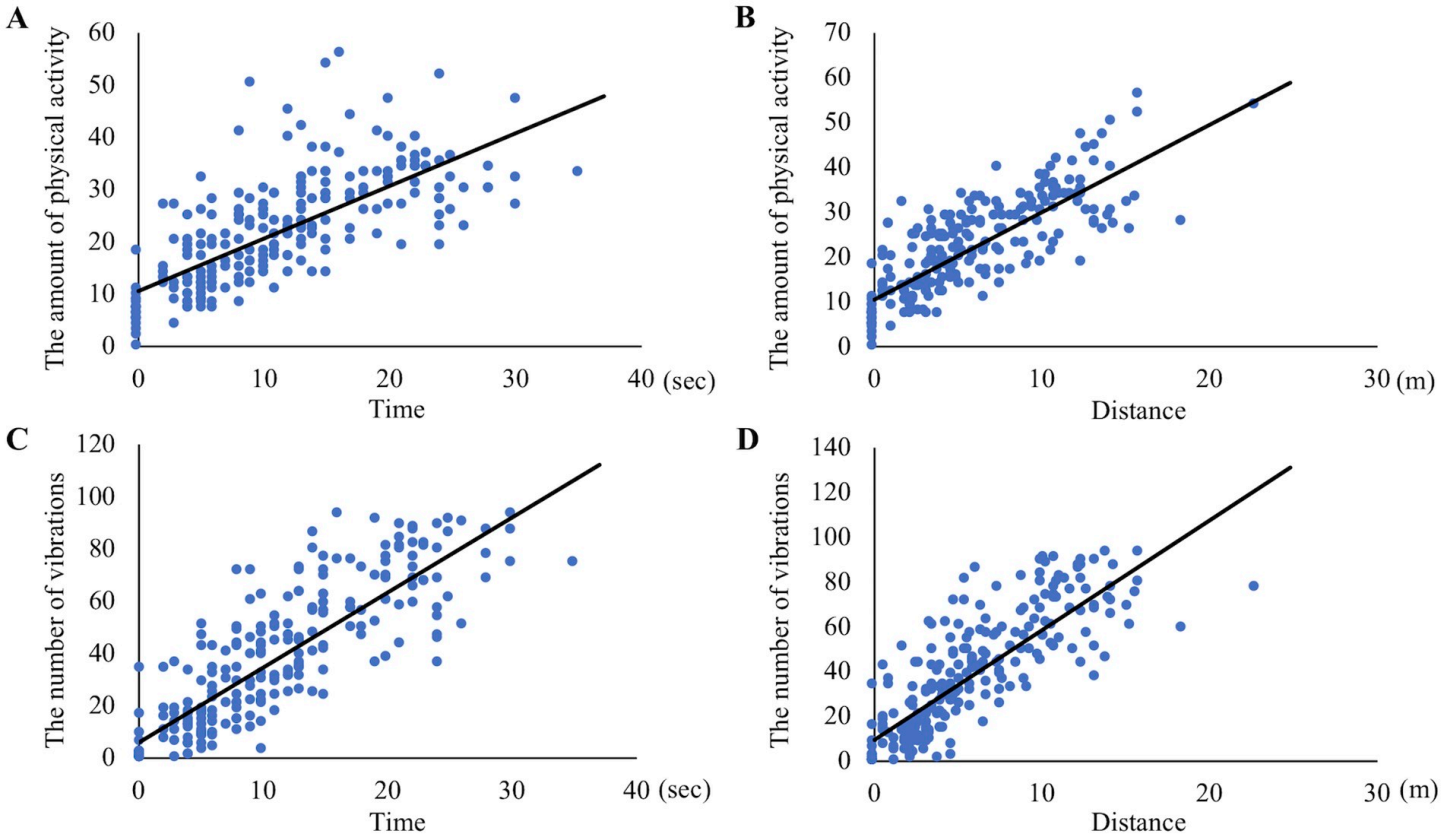
Correlations between physical activity data and moving time and distance. Plots showing correlations between the amount of physical activity and (A) moving time and (B) distance. Other plots showing correlations between the number of vibrations and (C) moving time and (D) distance.

**Fig 7:**
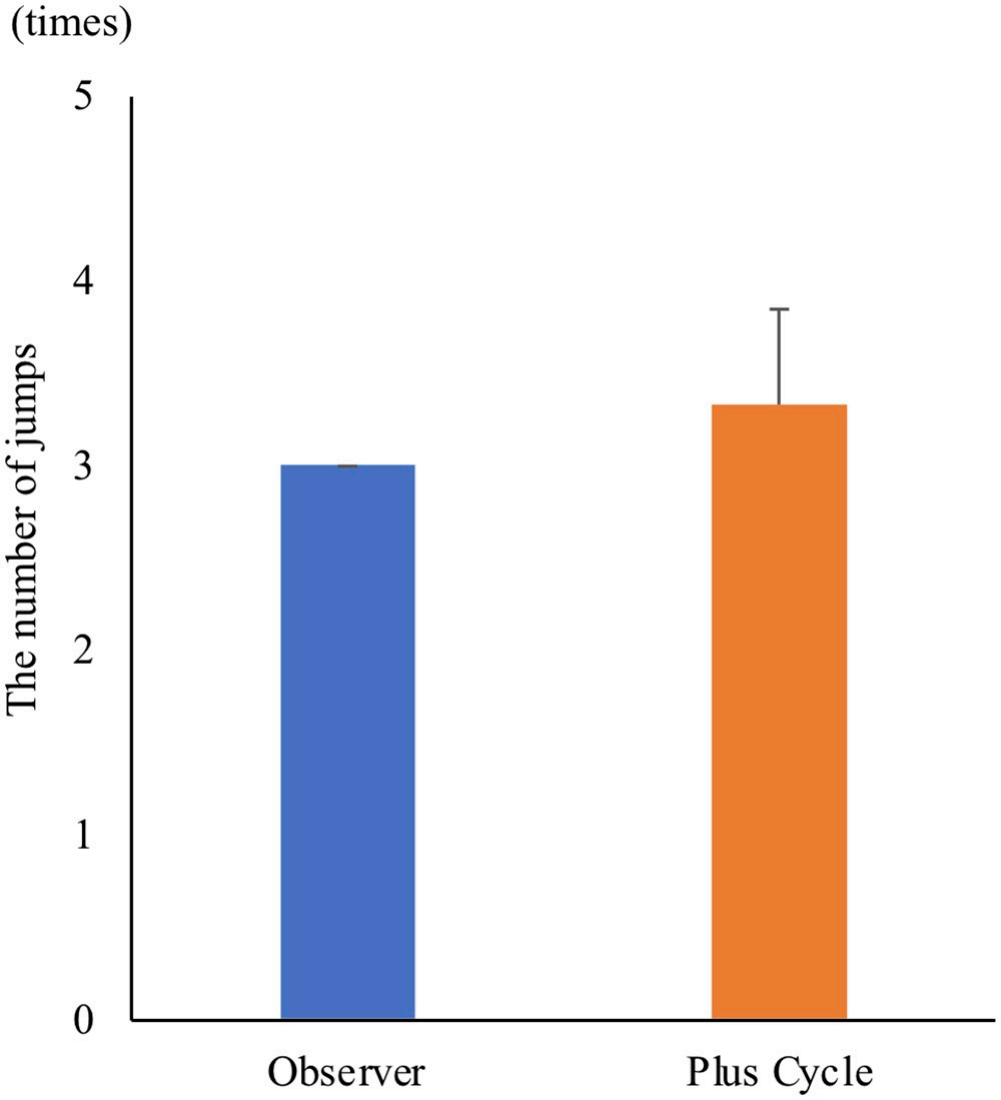
The number of jumps counted by the observer and Plus Cycle^®^. There was no significant difference between the number counted by observer and outputs of Plus Cycle^®^. The results of the number of jumps are presented as mean ± standard deviation.

**Fig 8:**
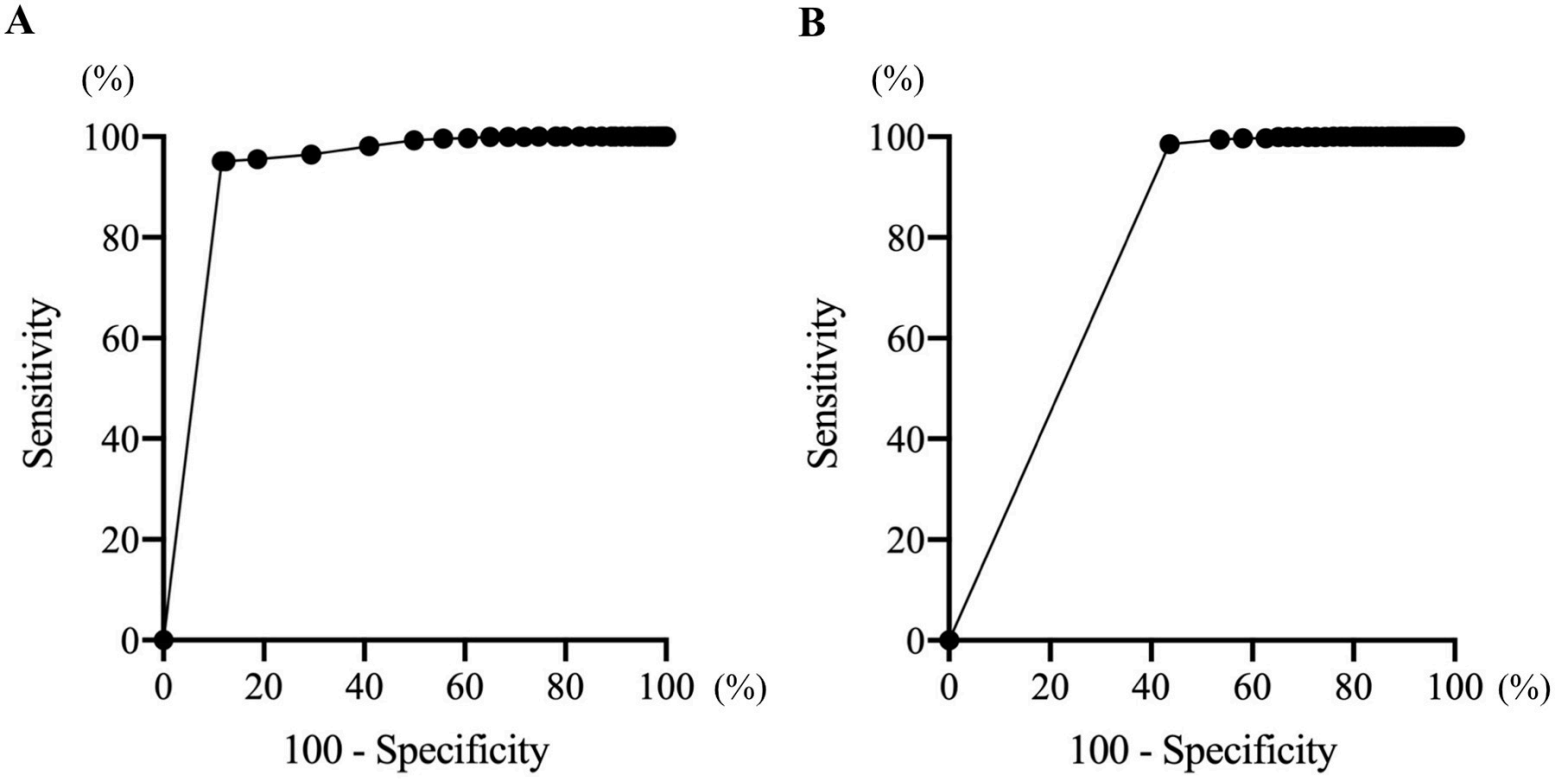
ROC curve showing the accuracy of measuring the resting and sleeping time. (A) ROC curve of the amount of physical activity and (B) ROC curve of the number of vibrations.

There were significant positive correlations between the amount of physical activity and moving time (p < 0.05, r = 0.72) and distance (p < 0.05, r = 0.80). In addition, significant positive correlations were also detected between the number of vibrations and moving time (p < 0.05, r = 0.84) and distance (p < 0.05, r = 0.82) (Fig 6). There was no significant difference between the number of actual jumps counted by the observer and that counted by Plus Cycle^®^ (Fig 7). The optimal cut-off value for the amount of physical activity to distinguish whether cats were resting and sleeping or awake was 0.5 per minute with sensitivity of 95.1%, specificity of 88.4% and AUC of 0.93. With regard to the number of vibrations, the optimal cut-off value was 0.5 per minute with sensitivity of 98.6%, specificity of 56.4% and AUC of 0.78 (Fig 8).

### Assessment of physical activities and sleep quality using Plus Cycle^®^ in client-owned cats

Sixty-one cats (31 males and 30 females) were included in the present study. The mean age was 7.6 ± 5.1 years (range, 1 to 19 years). Breeds included Japanese domestic cats (n = 47), Scottish Fold (n = 3), Japanese Bobtail (n = 2), Tonkinese (n = 2), American Bobtail (n = 1), Minuet (n = 1), Munchkin (n = 1), Norwegian Forest Cat (n = 1), Oriental (n = 1), Ragdoll (n = 1) and Russian Blue (n = 1).

**Fig 9:**
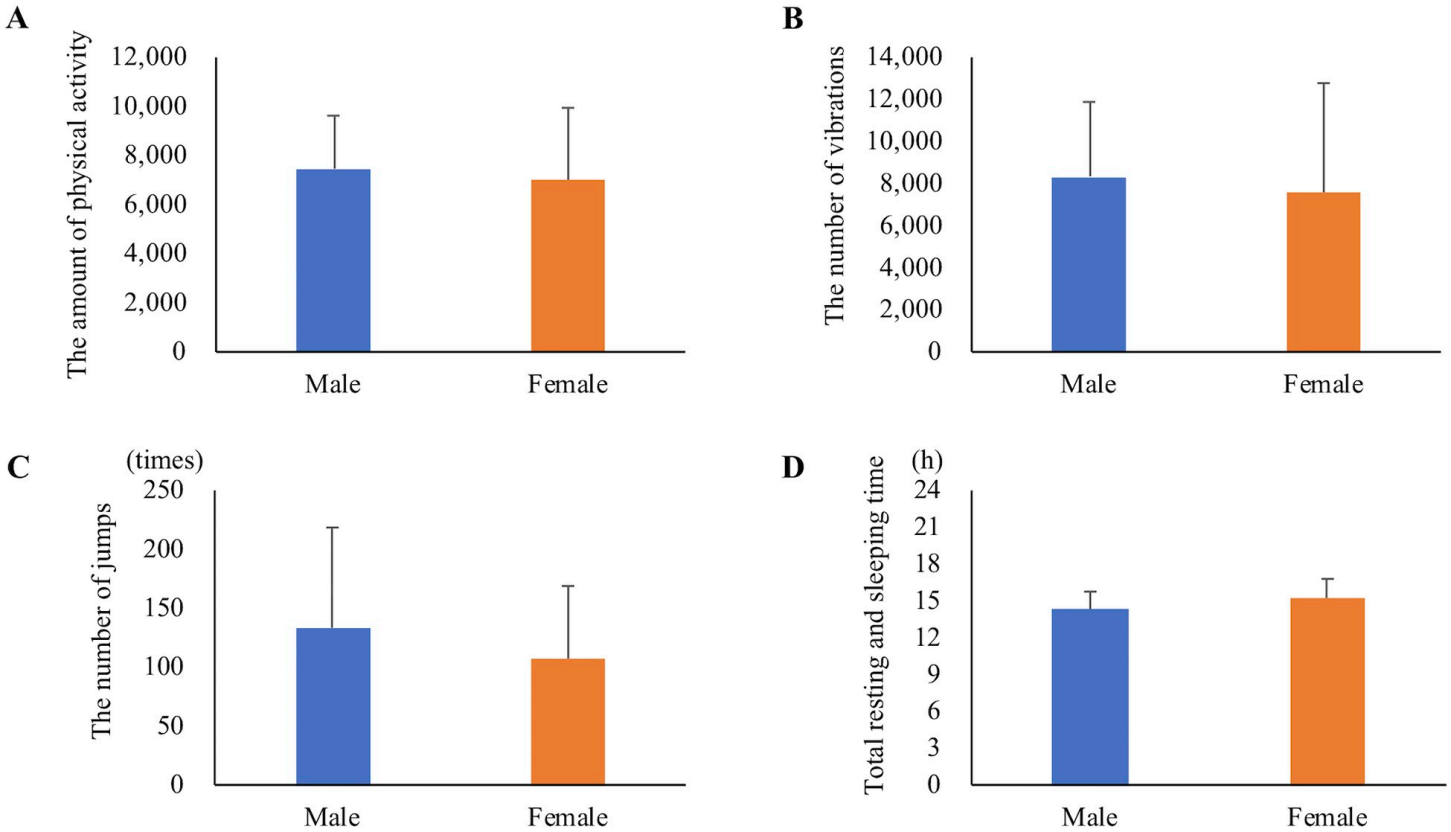
Difference in the data of physical activities and sleep quality by gender. (A) The amount of physical activity, (B) the number of vibrations, (C) the number of jumps, (D) total resting and sleeping time. All data are presented as mean ± standard deviation.

**Fig 10:**
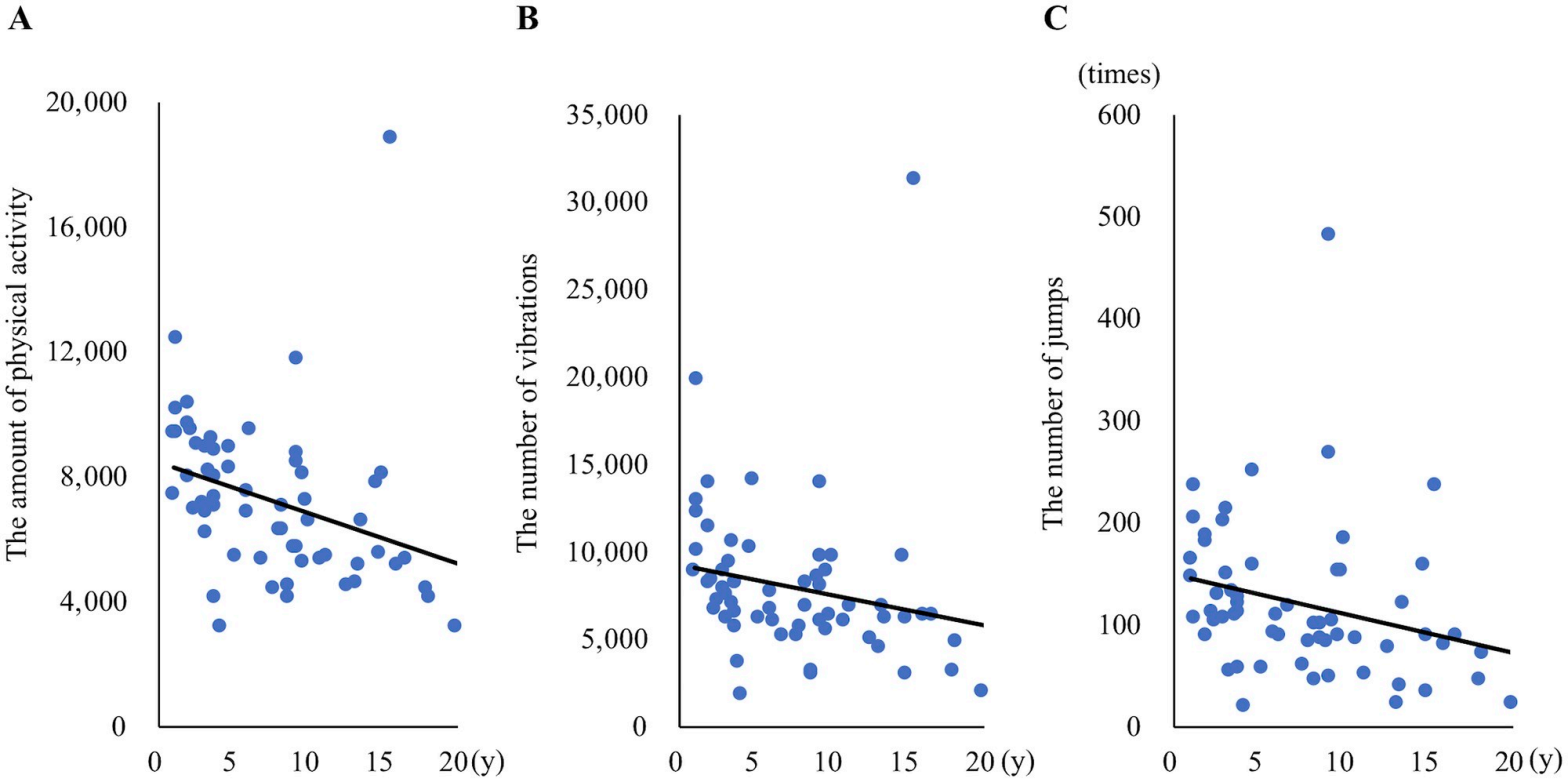
Correlation between physical activity data and age. Plots showing correlation between (A) the amount of physical activity, (B) the number of vibrations, (C) the number of jumps and age.

**Fig 11:**
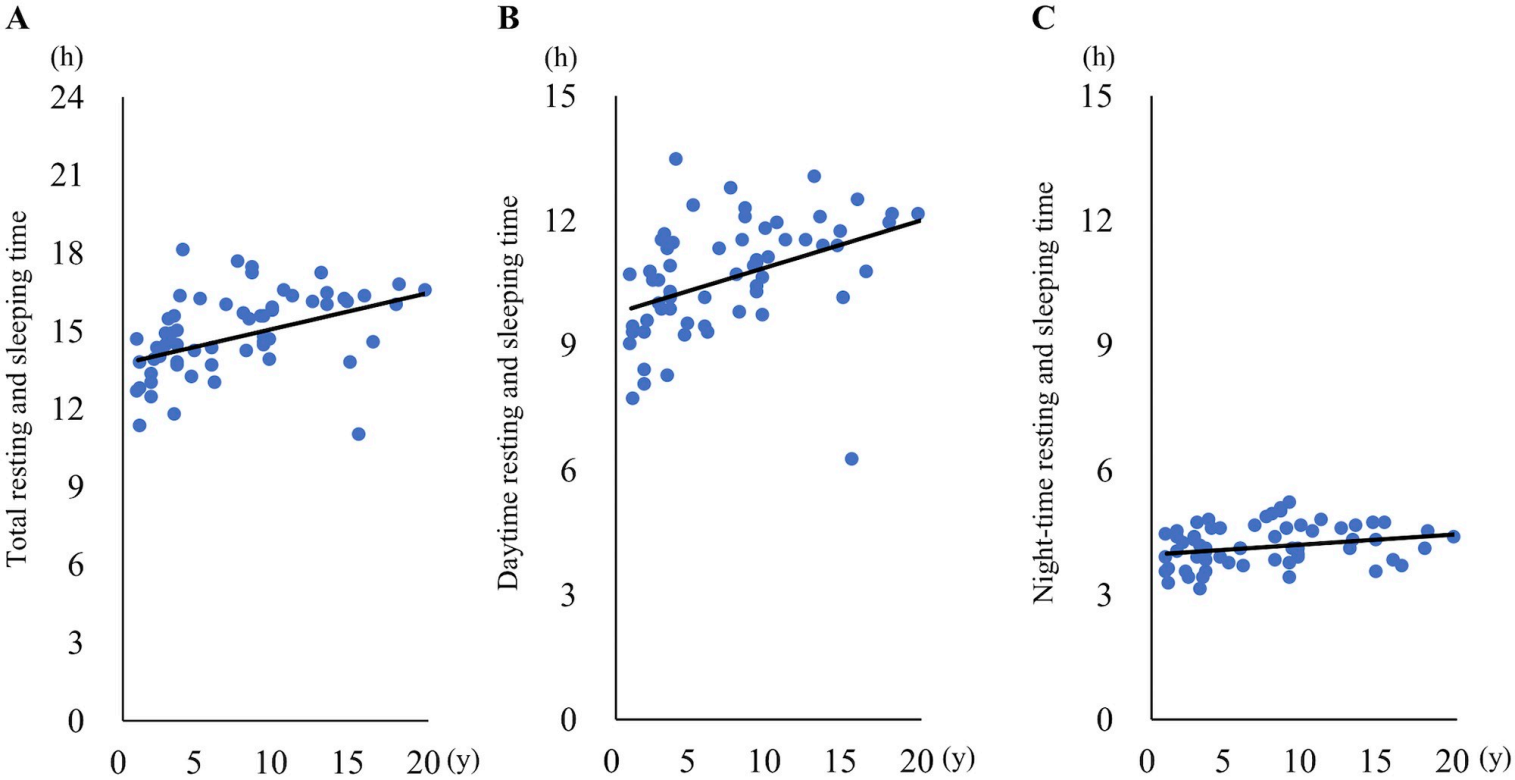
Correlation between resting and sleeping time and age. Plots showing correlation between (A) total resting and sleeping time, (B) daytime resting and sleeping time, (C) night-time resting and sleeping time and age.

When the daily average values were compared by gender, no significant differences were detected in the amount of physical activity, the number of vibrations, the number of jumps, and total resting and sleeping time (Fig 9). The daily average of the amount of physical activity (p < 0.05, r = -0.32), the number of vibrations (p = 0.12, r = -0.2) and the number of jumps (p < 0.05, r = -0.26) were correlated negatively with the age of the cats (Fig 10). On the other hand, there was a significant positive correlation between the daily average of total resting and sleeping time and age (p < 0.05, r = 0.45). In addition, a significant positive correlation was also detected between the daily average of daytime resting and sleeping time and age (p < 0.05, r = 0.41). Although there was a significant positive correlation between the daily average of night-time resting and sleeping time and age (p<0.05, r = 0.25), the change in the time with aging was very small, unlike total and daytime resting and sleeping time (Fig 11).

## Discussion

In the present study, there were strong correlations between our custom-developed Plus Cycle^®^ and Actical^®^ in total activity and activity intensity. When the amount of physical activity, the number of vibrations and the number of jumps were measured by using Plus Cycle^®^ in healthy cats, the physical activities were quantified with high accuracy. In addition, it was also found to be very accurate in discriminating resting and sleeping conditions of cats. In client-owned cats, there were no significant differences by gender in any measured traits. The amount of physical activity and the number of jumps significantly decreased with aging of cats. In contrast, the resting and sleeping times significantly increased with aging. The results of this study revealed that the change in the night-time resting and sleeping time with aging was very small.

Activity monitors, which can measure physical activities objectively, have been used to detect behavioral changes of cats in previous studies [7, 11, 15–18]. In most of those studies, Actical^®^, a human activity monitor, was applied to assess feline physical activities, and its measurement accuracy has also been validated already in cats [11]. In addition, Actical^®^ has been used to evaluate treatment efficacy of analgesics or non-steroidal anti-inflammatory drugs (NSAIDs) in cats with OA [7, 17, 18]. However, Actical^®^ cannot measure the number of jumps, which is the most common clinical sign in cats with OA, because this device is not equipped with air pressure sensor. In addition, an application to measure sleeping time is not included in the data analysis software of Actical^®^. Plus Cycle^®^ with built-in three-dimensional accelerometer and an air pressure sensor and an included application to measure resting and sleeping time was developed. In the present study, the data measured by Plus Cycle^®^ strongly correlated with the data measured by Actical^®^ that was used in many previous studies, and accurately reflected actual behavior of cats. These results suggest that, like Actical^®^, Plus Cycle^®^ can also be used to evaluate physical activities and to investigate the treatment efficacy of analgesics or NSAIDs in cats.

In cats, it has been reported that deterioration of joint components and functional decline occur with normal aging, even in the absence of musculoskeletal disorders [19]. However, to the best of our knowledge, there have been no studies that revealed objectively the changes in physical activities with aging in cats. In order to detect a decrease in physical activities due to musculoskeletal disorders such as OA, it is necessary to understand the normal age-related changes in physical activities in cats. The present study objectively demonstrated that not only the amount of physical activity but also the number of jumps significantly decreased with aging of cats. This data will be useful to distinguish the decrease in physical activities due to musculoskeletal disorders such as OA from age-related changes in cats.

It has been reported that chronic pain due to OA can lead to significant disruption of sleep in humans and dogs [14, 20]. A previous study reported that 81% of humans with hip or knee OA experienced night pain, which contributed to sleep disturbance [20]. It is reported that pain-associated sleep disturbance also occurs in dogs with OA [14]. In cats with OA, it is well known that behavioral changes such as resting more than usual and increased sleeping of daytime occur [4, 5]. To reveal whether the increased sleeping time of cats with OA is due to sleep disturbance or aging, sleeping time of daytime and night-time were investigated in client-owned cats. The present study revealed that daytime resting and sleeping time significantly increased with aging of cats. However, the age-related change in night-time resting and sleeping time quantified by Plus Cycle^®^ was very small. Therefore, objective evaluation of night-time sleep quality may also help in the early diagnosis of OA with chronic pain in cats.

The limitations of this study include the small numbers of cats in each age. Client-owned cats used in this study were not classified as healthy or diseased cats, and indoor or outdoor cats; cats of all backgrounds were tested. In addition, no data on cats with a definitive diagnosis of OA were obtained in the present study. Further studies such as the establishment of reference values for cats are needed to utilize Plus Cycle^®^ for diagnosis of feline OA.

## Conclusion

Plus Cycle^®^ can accurately and objectively assess physical activities and sleep quality in cats. This novel activity monitor has potential for use to monitor musculoskeletal health care of cats, especially since this device can measure the number of jumps and resting and sleeping times of cats.

## Supporting information

S1 Table Summary of the cats used for comparison of Plus Cycle^®^ with human activity monitor. (TIF)

S2 Table Summary of the cats used to test the validity of measurement accuracy of Plus Cycle. (TIF)
