# Supplementary figures and images for: Utility of a novel activity monitor assessing physical activities and sleep quality in cats
Source: PLoS One. 2020 Jul 31;15(7):e0236795. doi: 10.1371/journal.pone.0236795 (PMC7394395; doi:10.1371/journal.pone.0236795)

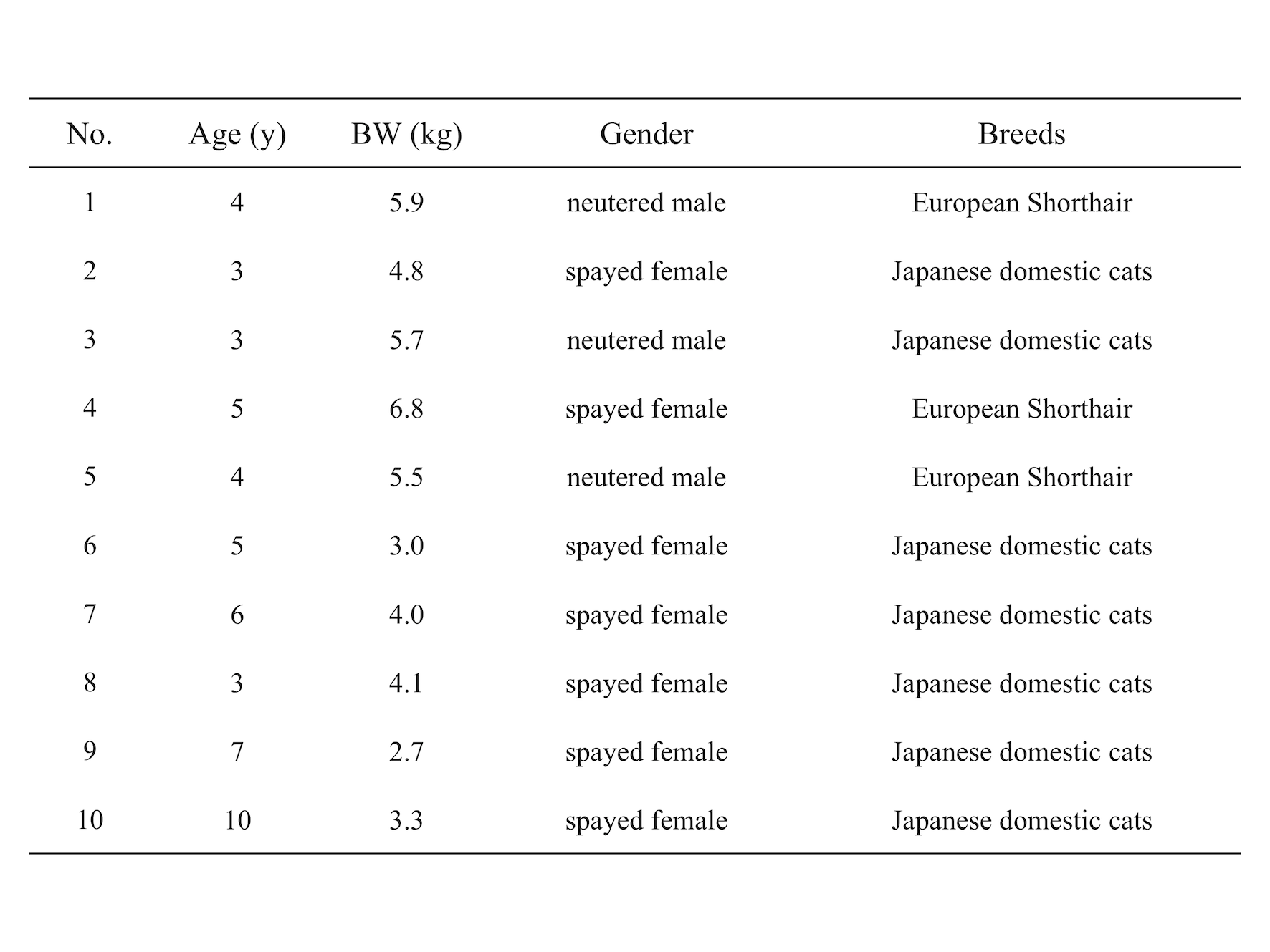

Supplement: S1 Table — (TIF) [file pone.0236795.s001.tif]

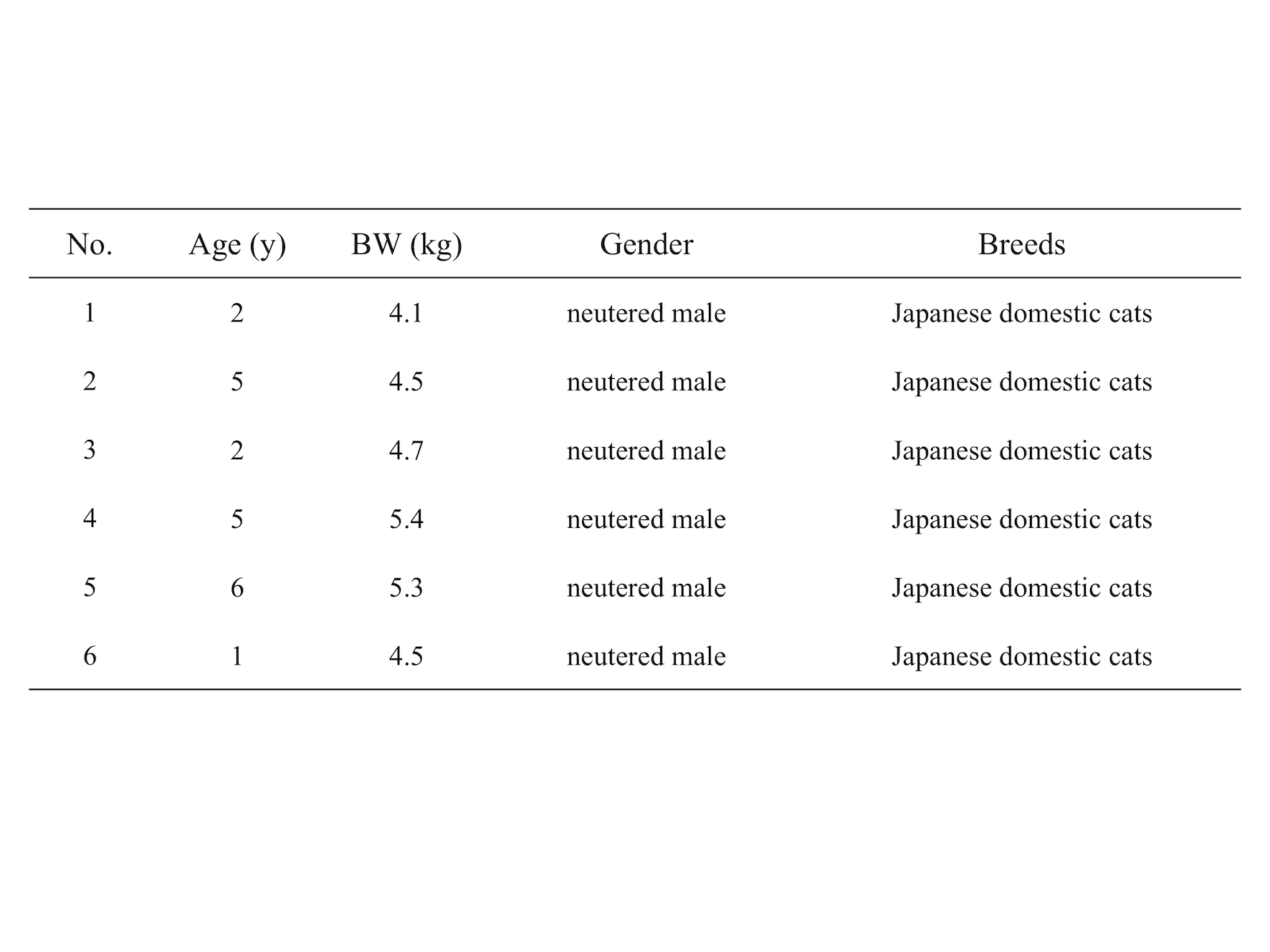

Supplement: S2 Table — (TIF) [file pone.0236795.s002.tif]
